# Antibacterial and antioxidant properties of the methanol extracts of the leaves and stems of *Calpurnia aurea*

**DOI:** 10.1186/1472-6882-8-53

**Published:** 2008-09-20

**Authors:** Adeolu A Adedapo, Florence O Jimoh, Srinivas Koduru, Anthony J Afolayan, Patrick J Masika

**Affiliations:** 1 Department of Veterinary Physiology, Biochemistry and Pharmacology, University of Ibadan, Ibadan, Nigeria; 2 Department of Botany, University of Fort Hare, Alice 5700, South Africa; 3 ARDRI, University of Fort Hare, Alice 5700, South Africa

## Abstract

**Background:**

In South Africa, *Calpurnia aurea* (Ait.) Benth is used to destroy lice and to relieve itches, to destroy maggots and to treat allergic rashes, particularly those caused by caterpillars. Antioxidants play an important role protecting against damage by reactive oxygen species. Plants containing flavonoids have been reported to possess strong antioxidant properties.

**Methods:**

The antibacterial, antioxidant activities and phenolic contents of the methanol extracts of the leaves and stems of *Calpurnia aurea* were evaluated using in vitro standard methods. Spectrophotometry was the basis for the determinations of total phenol, total flavonoids, flavonols, and proanthocyanidins. Tannins, quercetin and catechin equivalents were used for these parameters. The antioxidant activities of the stem extract of *Calpurnia aurea* were determined by ABTS, DPPH, and ferrous reducing antioxidant property (FRAP) methods. Laboratory isolates of 10 bacteria species which included five Gram-positive and five Gram-negative strains were used to assay for antibacterial activity of this plant.

**Results:**

The results from this study showed that the antioxidant activities of the stem extract of *Calpurnia aurea *as determined by the total phenol, flavonoids, and FRAP methods were higher than that of the leaves. On the other hand, the leaf extract of the plant has higher level of total flavonols and proanthocyanidins. The leaf extract also has higher radical scavenging activity as shown in 1, 1-Diphenyl-2-picrylhydrazyl (DPPH), and 2,2¿-azinobis-3- ethylbenzothiazoline-6-sulfonic acid (ABTS) assay. The leaf extract showed activity against seven of the bacterial organisms.

**Conclusion:**

The results from this study indicate that the leaves and stem extracts of *Calpurnia aurea *possess antioxidant properties and could serve as free radical inhibitors or scavenger or, acting possibly as primary antioxidants. Although, the antibacterial properties of *Calpurnia aurea* are not as effective as the standard drugs- Chloramphenicol and Streptomycin, they still possess some activity against bacterial strains used in this study. *Calpurnia aurea *may therefore be a good candidate for functional foods as well as pharmaceutical plant-based products.

## Background

The genus *Calpurnia *(Leguminosae) comprises some seven species which are widely distributed in South Africa. *Calpurnia aurea *(Ait.) Benth. is a yellow-flowered small tree or shrub (Natal Laburnum) widely distributed in Africa from Cape Province to Eritrea and which also occurs in Southern India [1].

Chemical investigations of *C. aurea *have resulted in the isolation of a series of quinolizidine alkaloids. The leaves and twigs of Ethiopian *C. aurea *yielded 13-hydroxylupanine. The South African species yielded the well known alkaloids: hydroxylupanine, calpurnine, virgiline and its pyrrolylcarboxylic acid ester as found in Ethiopian sample. In addition, the alkaloid 10, 13-dihydroxylupanine was found in CH_2_Cl_2 _extract of the pods. This compound having a MW of 280 and also occurring in *Cadia purpurea *was absent from the Ethiopian species. Two alkaloids (calpurmenine and 13-2'-pyrrolylcarboxyl) calpurmenine), specifically present in the South African material were isolated from the extracts of leaves and pods [1-3].

*Calpurnia aurea *is used for the treatment of amoebic dysentery and diarrhea in animals, killing head lice in humans and ticks in animals, syphilis, diarrhea, leishmaniasis, tapeworm, trachoma, *Tinea capitis*, wound, scabies, elephantiasis and different swellings [4-7]. In South Africa, Calpurnia leaves and powdered roots are used to destroy lice and to relieve itches. Unspecified parts are used to destroy maggots and the leaves are used to treat allergic rashes, particularly those caused by caterpillars. In East Africa, leaf sap is used to destroy maggots in wounds. In Nigeria, the seeds are used to treat abscesses. In Ethiopia it is used to treat stomach complaints, headache, eye diseases, amoebic dysentery, scabies (skin infection caused by ticks) and as an insecticide [7,8].

Free radicals have been implicated in the causation of several diseases such as liver chirrhosis, atherosclerosis, cancer, diabetes, etc. and compounds that can scavenge free radicals have great potential in ameliorating these disease processes [9-14]. Antioxidants thus play an important role to protect the human body against damage by reactive oxygen species [15,16]. Free radicals or reactive oxygen species (ROS) are produced *in vivo *from various biochemical reactions and also from the respiratory chain as a result of occasional leakage. These free radicals are the main culprits in lipid peroxidation [17]. Plants containing flavonoids have been reported to possess strong antioxidant properties [18,19].

Natural products from microorganisms have been the primary source of antibiotics, but with the increasing acceptance of herbal medicine as an alternative form of health care, the screening of medicinal plants for active compounds has become very important because these may serve as promising sources of novel antibiotic prototypes [20-22]. It has been shown that *in vitro *screening methods could provide the needed preliminary observations necessary to select crude plant extracts with potentially useful properties for further chemical and pharmacological investigations [23].

In the present study, the methanol extracts of the leaves and stem of *Calpurnia aurea *were screened for antioxidant and antibacterial properties using standard methods. The findings from this work may add to the overall value of the medicinal potential of the herb.

## Methods

### Plant collection

The stems and leaves of *Calpurnia aurea *were collected July 2006 in the Eastern Cape Province of South Africa. The area falls within the latitudes 30°00–34° 15'S and longitudes 22° 45'–30° 15'E. It is bounded by the sea in the east and the drier Karoo (semi-desert vegetation) in the west [24]. These areas consist of villages which are generally classified as rural and poor. The plants were identified by their vernacular names and later validated at the Department of Botany, University of Fort Hare by Professor Grierson and voucher specimens (Aded Med 2007/1–10) were deposited in the Griffen Herbarium of the University.

### Extract preparation

Both stems and leaves were air dried at room temperature to constant weights. The dried plant materials were separately ground to powders. Two hundred grams of powdered leaves and stem were soaked in 1 L of methanol separately for 48 hrs on an orbital shaker. Extracts were filtered using a Buckner funnel and Whatman No 1 filter paper. Each filtrate was concentrated to dryness under reduced pressure at 40°C using a rotary evaporator. The percentage yield for the leaves was 8.3% while that of the stems was 7.8%. Each extract was resuspended in methanol to make a 50 mg/ml stock solution [25].

### Chemicals

1,1-Diphenyl-2-picrylhydrazyl (DPPH), 2,2'-azinobis-3- ethylbenzothiazoline-6-sulfonic acid (ABTS), 3-(2-pyridyl)-5,6-diphenyl-1,2,4-triazine-4',4"-disulfonic acid, potassium ferricyanide; catechin, butylated hydroxytoluene (BHT), ascorbic acid, catechin, tannic acid, quercetin and FeCl_3 _were purchased from Sigma Chemical Co. (St. Louis, MO, USA)., vanillin from BDH; Folin-Ciocalteus's phenol reagent and sodium carbonate were from Merck Chemical Supplies (Damstadt, Germany). All the other chemicals used including the solvents, were of analytical grade.

### Determination of total phenolics

Total phenol contents in the extracts were determined by the modified Folin-Ciocalteu method [26]. An aliquot of the extract was mixed with 5 ml Folin-Ciocalteu reagent (previously diluted with water 1:10 v/v) and 4 ml (75 g/l) of sodium carbonate. The tubes were vortexed for 15 sec and allowed to stand for 30 min at 40°C for color development. Absorbance was then measured at 765 nm using the Hewlett Packard UV-VS spectrophotometer. Samples of extract were evaluated at a final concentration of 0.1 mg/ml. Total phenolic content were expressed as mg/g tannic acid equivalent using the following equation based on the calibration curve: y = 0.1216x, R^2 ^= 0.9365, where x was the absorbance and y was the tannic acid equivalent (mg/g).

### Determination of total Flavonoids

Total flavonoids were estimated using the method of Ordon ez et al. [27]. To 0.5 ml of sample, 0.5 ml of 2% AlCl_3 _ethanol solution was added. After one hour at room temperature, the absorbance was measured at 420 nm. A yellow color indicated the presence of flavonoids. Extract samples were evaluated at a final concentration of 0.1 mg/ml. Total flavonoid content were calculated as quercetin (mg/g) using the following equation based on the calibration curve: y = 0.0255x, R^2 ^= 0.9812, where x was the absorbance and was the quercetin equivalent (mg/g).

### Determination of total Flavonols

Total flavonols in the plant extracts were estimated using the method of Kumaran and Karunakaran [28]. To 2.0 mL of sample (standard), 2.0 mL of 2% AlCl_3 _ethanol and 3.0 mL (50 g/L) sodium acetate solutions were added. The absorption at 440 nm was read after 2.5 h at 20°C. Extract samples were evaluated at a final concentration of 0.1 mg/ml. Total flavonoid content was calculated as quercetin (mg/g) using the following equation based on the calibration curve: y = 0.0255x, R^2 ^= 0.9812, where x was the absorbance and was the quercetin equivalent (mg/g).

### Determination of total proanthocyanidins

Determination of proanthocyanidin was based on the procedure reported by Sun et al. [29]. A volume of 0.5 ml of 0.1 mg/ml of extract solution was mixed with 3 ml of 4% vanillin-methanol solution and 1.5 ml hydrochloric acid; the mixture was allowed to stand for 15 min. The absorbance was measured at 500 nm. Extract samples were evaluated at a final concentration of 0.1 mg/ml. Total proanthocyanidin content were expressed as catechin equivalents (mg/g) using the following equation based on the calibration curve: y = 0.5825x, R^2 ^= 0.9277, where x was the absorbance and y is the catechin equivalent (mg/g).

### Determination of antioxidant activity

#### ABTS radical scavenging assay

For ABTS assay, the method of Re et al. [30] was adopted. The stock solutions included 7 mM ABTS solution and 2.4 mM potassium persulfate solution. The working solution was then prepared by mixing the two stock solutions in equal quantities and allowing them to react for 12 h at room temperature in the dark. The solution was then diluted by mixing 1 ml ABTS^.+ ^solution with 60 ml methanol to obtain an absorbance of 0.706 ± 0.001 units at 734 nm using the spectrophotometer. Fresh ABTS solution was prepared for each assay. Plant extracts (1 ml) were allowed to react with 1 ml of the ABTS solution and the absorbance was taken at 734 nm after 7 min using the spectrophotometer. The ABTS^.+ ^scavenging capacity of the extract was compared with that of BHT and percentage inhibition calculated as ABTS radical scavenging activity (%) = [(Abs_control _- Abs_sample_)]/(Abs_control)_] × 100 where Abs_control _is the absorbance of ABTS radical + methanol; Abs_sample _is the absorbance of ABTS radical + sample extract/standard.

#### DPPH radical scavenging assay

The effect of extracts on DPPH radical was estimated using the method of Liyana-Pathirana and Shahidi [31]. A solution of 0.135 mM DPPH in methanol was prepared and 1.0 ml of this solution was mixed with 1.0 ml of extract in methanol containing 0.02–0.1 mg of the extract. The reaction mixture was vortexed thoroughly and left in the dark at room temperature for 30 min. The absorbance of the mixture was measured spectrophotometrically at 517 nm. Ascorbic acid and BHT were used as references. The ability to scavenge DPPH radical was calculated by the following equation: DPPH radical scavenging activity (%) = [(Abs_control _- Abs_sample_)]/(Abs_control)_] × 100 where Abs_control _is the absorbance of DPPH radical + methanol; Abs_sample _is the absorbance of DPPH radical + sample extract/standard.

#### Total antioxidant activity (FRAP assay)

A modified method of Benzie and Strain [32] was adopted for the FRAP assay. The stock solutions included 300 mM acetate buffer (3.1 g C_2_H_3_NaO_2_·3H_2_O and 16 ml C_2_H_4_O_2_), pH 3.6, 10 mM TPTZ (2, 4, 6-tripyridyl-*s*-triazine) solution in 40 mM HCl, and 20 mM FeCl_3_·6H_2_O solution. The fresh working solution was prepared by mixing 25 ml acetate buffer, 2.5 ml TPTZ, and 2.5 ml FeCl_3_·6H_2_O. The temperature of the solution was raised to 37°C before using. Plant extracts (150 μL) were allowed to react with 2850 μl of the FRAP solution for 30 min in the dark condition. Readings of the colored product (ferrous tripyridyltriazine complex) were taken at 593 nm. The standard curve was linear between 200 and 1000 μM FeSO_4_. Results were expressed in μM Fe (II)/g dry mass and compared with that of BHT, ascorbic acid and catechin.

#### Bioassay

Laboratory isolates of 10 bacteria species which included five Gram-positive and five Gram-negative strains were obtained from the Department of Biochemistry and Microbiology, Rhodes University, South Africa. The Gram-positive strains were *Bacillus cereus*, *Staphylococcus epidermidis*, *Staphylococcus aureus*, *Micrococcus kristinae*, and *Streptococcus pyogens*). The five Gram- negative strains were *Escherichia coli*, *Salmonella pooni*, *Serratia marcescens*, *Pseudomonas aeruginosa*, and *Klebsiella pneumoniae*. Each organism was maintained on nutrient agar slants and was recovered by sub-culturing in nutrient broth (Biolab No. 2) for 24 hrs. Before use, each bacterial culture was diluted 1:100 with fresh sterile nutrient broth [33].

Test organisms were streaked in a radial pattern on sterile nutrient agar plates and incubated at 37°C and examined after 24 and 48 hrs. Complete suppression of growth by a specific concentration of an extract was required to be declared active. Each extract was tested at final concentrations of 0.1, 0.5, 1.0, 2.5 and 5.0 mg/ml [22,34]. Blank plates containing only nutrient agar, and other sets containing nutrient agar and methanol served as controls while Chloramphenicol and Streptomycin served as standards. Each treatment was performed in triplicate and complete inhibition of bacterial growth was required for an extract to be declared bioactive.

### Statistical analysis

The experimental results were expressed as mean ± standard error of mean (SEM) of three replicates. Where applicable, the data were subjected to one way analysis of variance (ANOVA) and differences between samples were determined by Duncan's Multiple Range test using the Statistical Analysis System (SAS, 1999) program. *P *values < 0.05 were regarded as significant and *P *values < 0.01 as very significant.

## Results

### Total phenolic, flavonoids and proanthocyanidin contents

Results obtained in the present study revealed that the level of these phenolic compounds in the methanol extracts of the leaves and stem of *C. aurea *were considerable. The stem extract higher levels of total phenol and flavonoids than the leaf extract. On the other hand, the leaf extract possessed higher levels of proanthocyanidins and total flavonols (Table 1).

**Table 1:** Polyphenol contents of the methanol extracts of the leaves and stems of *Calpurnia aurea*. (n = 3, X ± SEM).

Phenolics	Leaves	Stems
Total polyphenol^a^	9.62 ± 0.53	11.79 ± 0.14*
Flavonoids^b^	0.81 ± 0.02	1.11 ± 0.02*
Total Flavonol^c^	0.83 ± 0.10*	0.10 ± 0.02
Proanthocyanidins^d^	4.37 ± 0.32*	1.10 ± 0.04

^a^Expressed as mg tannic acid/g of dry plant material.

^b^Expressed as mg quercetin/g of dry plant material.

^c^Expressed as mg quercetin/g of dry plant material.

^d^Expressed as mg quercetin/g of dry plant material

* indicates that this value is significantly different from the other at P < 0.05

### Total antioxidant power (FRAP)

The reducing ability of the extracts was in the range of 111.49 – 3146.89 μm Fe (II)/g (Table 2). The FRAP values for the extracts were significantly lower than that of ascorbic acid, quercetin and catechin, but higher than that of BHT.

**Table 2:** Total antioxidant activity of the leaves and stem extracts of *Calpurnia aurea*

Extracts	FRAP
Leaves	111.98 ± 38.89
Stem	3146.98 ± 63.67
Ascorbic acid	1632.1 ± 16.95
BHT	63.46 ± 2.49
Catechin	972.02 ± 0.61
Quercetin	3107.29 ± 31.28

### DPPH radical scavenging activity

**Figure 1:**
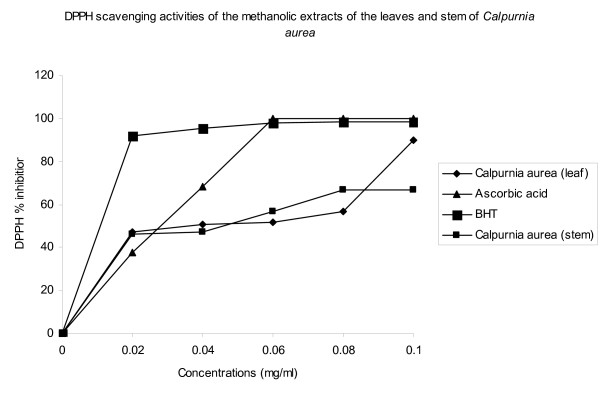
DPPH scavenging activities of the methanolic extracts of the leaves and stem of *Calpurnia aurea*

Figure 1 shows the dose-response curve of DPPH radical scavenging activity of the methanolic extracts of the leaves and stem of *C. aurea*, compared with BHT and ascorbic acid. It was observed that extract of the leaves had higher activity than that of the stem. At a concentration of 0.1 mg/ml, the scavenging activity of the leaves reached 89.7%, while at the same concentration, that of the stem was 67.0%.

### ABTS radical scavenging activity

**Figure 2:**
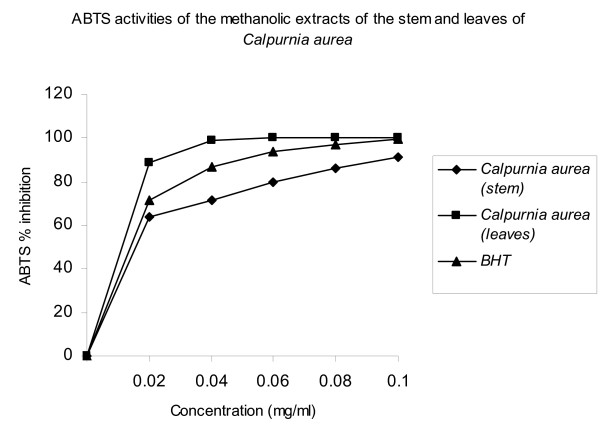
ABTS scavenging activities of the methanolic extracts of the leaves and stem of *Calpurnia aurea*.

The methanol extracts of the leaves and stem of *C. aurea *were fast and effective scavengers of the ABTS radical (Fig 2) and this activity was comparable to that of BHT. The percentage inhibition was 100, 98.8 and 99.3% for the leaf, stem and BHT respectively.

### Antibacterial activity

The antibacterial activity of the extracts of the leaves and stem of the plant is presented in Table 3. The antibacterial activity of the methanol extracts of the leaves of the *C. aurea *is much higher than of the stem. The leaf extract also has activity against all the organisms except *Serratia marcescens, Pseudomonas aeruginosa *and *Klebsiella pneumoniae *at MIC of 5 mg/ml while that of the stem was only active against *Bacillus cereus *and *Streptococcus pyrogens *at similar concentrations.

**Table 3:** Antibacterial activity of the leaves and stem extracts of *Calpurnia aurea*.

		Minimum inhibitory concentration (mg/ml)			
Bacterial species	Gram +/-	Leaves	Stem	Chloramphenicol μg/ml	Streptomycin μg/ml

*Bacillus cereus*	+	5.0	5.0	< 2	< 2
*Staphylococcus epidermidis*	+	5.0	na	< 2	< 2
*Staphylococcus aureus*	+	5.0	na	< 2	< 2
*Micrococcus kristinae*	+	5.0	na	< 2	< 2
*Streptococcus pyogens*	+	5.0	5.0	< 2	< 2
*Escherichia coli*	-	5.0	na	< 2	< 2
*Salmonella pooni*	-	5.0	na	< 2	< 2
*Serratia marcescens*	-	na	na	< 2	< 2
*Pseudomonas aeruginosa*	-	na	na	< 20	< 5
*Klebsiella pneumonae*	-	na	na	< 2	< 2

na = not active.

## Discussion

### Total phenolic, flavonoids and proanthocyanidin contents

Polyphenols are the major plant compounds with antioxidant activity. This activity is believed to be mainly due to their redox properties [35], which play an important role in adsorbing and neutralizing free radicals, quenching singlet and triplet oxygen, or decomposing peroxides. Results obtained in the present study revealed that the level of these phenolic compounds in the methanol extracts of the leaves and stem of *C. aurea *were considerable (Table 1). The results strongly suggest that phenolics are important components of this plant, and some of its pharmacological effects could be attributed to the presence of these valuable constituents.

### Total antioxidant power (FRAP)

The antioxidant potentials of the methanol extracts of the leaves and stem of *C. aurea *were estimated from their ability to reduce TPRZ-Fe (III) complex to TPTZ-Fe (II). The reducing ability of the extracts was in the range of 111.49 – 3146.89 μm Fe (II)/g (Table 2). Antioxidant activity increased proportionally with the polyphenol content. According to recent reports, a highly positive relationship between total phenols and antioxidant activity appears to be the trend in many plant species [36].

### DPPH radical scavenging activity

The effect of antioxidants on DPPH is thought to be due to their hydrogen donating ability [38]. Though the DPPH radical scavenging abilities of the extracts were significantly less than those of ascorbic acid (100%) and BHT (98.3), the study showed that the extracts have the proton-donating ability and could serve as free radical inhibitors or scavengers, acting possibly as primary antioxidants.

### ABTS radical scavenging activity

Proton radical scavenging is an important attribute of antioxidants. ABTS, a protonated radical, has characteristic absorbance maxima at 734 nm which decreases with the scavenging of the proton radicals [37]. The methanol extracts of the leaves and stem of *C. aurea *were fast and effective scavengers of the ABTS radical (Fig 2) and this activity was comparable to that of BHT. Higher concentrations of the extracts were more effective in quenching free radicals in the system.

The scavenging of the ABTS radical by the extracts was found to be much higher than that of DPPH radical. Factors like stereoselectivity of the radicals or the solubility of the extract in different testing systems have been reported to affect the capacity of extracts to react and quench different radicals [39]. Wang et al. [40] found that some compounds which have ABTS scavenging activity did not show DPPH scavenging activity. In this study, the extracts showed strong scavenging activities against DPPH and ABTS radicals. This further showed the capability of the extracts to scavenge different free radicals in different systems, indicating that they may be useful therapeutic agents for treating radical-related pathological damage.

### Antibacterial activity

The antibacterial activity of the methanol extracts of the leaves of the *C. aurea *is much higher than of the stem. It was observed that *Calpurnia aurea *showed strong antibacterial activity comparable to that of standard gentamycin (0.1 mg/ml) though at MIC of 100 mg/ml [7]. Though the minimum inhibitory concentration of 5 mg/ml is very high, nevertheless it showed that the plant extracts under *in vitro *study has broad spectrum antibacterial activity. It is known that, in general, the Gram-negative bacteria are more resistant than the Gram-positive ones [41,42], however, the study showed that 2 of the Gram-negative organisms used in this study were sensitive to this extract even then at high MIC of 5 mg/ml. *Streptococcus pyogens *is a known pathogen of respiratory infections [43]; its inhibition by the two extracts might suggest their possible use in the treatment of chest and respiratory infections. Furthermore, *Escherichia coli*, which is a Gram-negative bacterium, was also inhibited by the leave extract. Although it belongs to the normal flora of humans, an enterohaemorrhagic strain of *E. coli *has caused serious food poisoning, and preservatives to eliminate its growth are needed [44,45]; the extracts of *C. aurea *might therefore be of use.

## Conclusion

The results from this study indicate that the leaves and stem extracts of *Calpurnia aurea *possess antioxidant properties and could serve as free radical inhibitors or scavenger or, acting possibly as primary antioxidants. The antibacterial properties of *Calpurnia aurea *are not as effective as the standard drugs- Chloramphenicol and Streptomycin, but microorganisms become resistant to antibiotics over time. Again, of recent, a lot of attention is being devoted to natural sources of antioxidant and antibacterial materials, the data obtained in this study might suggest a possible use of *Calpurnia aurea *as a source of natural antioxidant and antimicrobial agents.

## Competing interests

The authors declare that they have no competing interests.

## Authors' contributions

AAA: Prepare the extract, carried out the assays and drafted the manuscript.

FOJ: Carried out the assay

SK: Carried out the bioassay

AJA: Coordinated the study

PJM: Provided the grants for the study and also coordinated the study.

All authors read and approved the final manuscript.

## Pre-publication history

The pre-publication history for this paper can be accessed here:


